# A single-center randomized controlled study of intraperitoneal hyperthermic chemoperfusion in combination of recombinant human tumor necrosis factor (TNF) in treatment of malignant ascites caused by advanced abdominal cancers

**DOI:** 10.1097/MD.0000000000031883

**Published:** 2022-12-02

**Authors:** Lexia Wu, Wanshan Zhu, Jincheng Meng, Jiaming Wu, Luzhen Li, Cantu Fang, Huatang Zhang

**Affiliations:** Zhongshan Affiliated Hospital, Guangzhou University of Chinese Medicine, Zhongshan, China.

**Keywords:** advanced gynecologic cancers, cisplatin, HIPEC, peritoneal effusion, rmhTNF-NC

## Abstract

Malignant peritoneal effusion is a common complication of advanced malignancies, which has a poor prognosis for patients. Hyperthermic intraperitoneal chemotherapy (HIPEC) has been widely used in the treatment of advanced gynecological tumors, especially ovarian cancer (OC). Relative studies have indicated that HIPEC allows for direct exposure of tumor cells to high peritoneal concentrations of cytotoxic drugs without increasing systemic toxicity compared with intravenous treatment. Recombinant human tumor necrosis factor for injection (rmhTNF-NC) is a safely tolerated immunotherapeutic drug that has becoming a mainstay of treatment for malignant effusions. Currently, a prospective study is required to determining the efficacy of rmhTNF-NC plus cisplatin for the treatment of malignant peritoneal effusion for OC.

**Methods:**

Design and setting: This is a single-center, open trial will be performed in Zhongshan Affiliated Hospital, Guangzhou University of Chinese Medicine.

**Participants:**

Eligible patients will be those with advanced gynecologic cancers and who would be suitable for HIPEC.

**Intervention and Control:**

HIPEC with cisplatin and intraperitoneal perfusion with rmhTNF-NC.

**Co-interventions:**

Further chemotherapy will be offered to patients as per current practice.

Outcomes

Pilot study: Patients and clinicians’ acceptability of the trial to assist in optimization of recruitment.

Primary outcome: One-year overall survival (OS).

Secondary outcomes: Progression-free survival (PFS), adverse events.

Follow-up: One-year follow-up for OS.

Sample size: Twenty patients to demonstrate therapeutic effect of peritoneal effusion caused by OC.

**Discussion:**

This trial will determine the effectiveness of HIPEC with cisplatin and intraperitoneal perfusion with rmhTNF-NC for advanced gynecologic cancers, and guide the optimal treatment for these patients.

## 1. Introduction

According to Global Cancer Statistics 2020, ovarian cancer (OC) is one of the most commonly diagnosed cancer (1.6% of total cases) and one of the leading cause of cancer death (2.1% of the total cancer deaths).^[1]^ OC is the leading cause of mortality among patients bearing gynaecological cancers and the majority of patients are diagnosed in advanced stages.^[2]^ At present, the standard first-line treatment for OC is cytoreductive surgery followed by platinum-based combination chemotherapy, which is basically the same as fallopian tube or primary peritoneal cancer.^[3–5]^ Malignant ascites refers to peritoneal effusion caused by primary peritoneal or other malignant tumors that metastasize to the peritoneum.^[6]^ Malignant peritoneal effusion is common in OC, and about 10% of OC patients are faced with recurrence and metastasis. Tumor cells in OC-associated malignant ascites promote disease recurrence and patient mortality is mainly associated with widespread metastasis to serosal surfaces and accompanying peritoneal effusions.^[7]^

There is no consensus on systematic treatment of malignant ascites in patients with advanced OC.^[8]^ Hyperthermic intraperitoneal chemotherapy (HIPEC) has been widely used in the treatment of advanced OC. At the same time, recombinant human tumor necrosis factor for injection (rmhTNF-NC) has been proved to be effective in treating thoracic and abdominal effusion caused by advanced tumors by a number of studies. In addition, there is no study, either prospective or retrospective, involving HIPEC combined with rmhTNF-NC in the treatment of abdominal effusion caused by advanced OC, and therefore, we designed a single-arm phase I trial to explore the efficacy of it.

## 2. Design

This clinical trial is a single-center exploratory single-arm study approved by the Ethics Review Committee of Zhongshan Affiliated Hospital, Guangzhou University of Chinese Medicine (Approval Number: 2021ZSZY-LLK-163). The trial is registered at chictr.org.cn (ChiCTR2100045155, May 7, 2021). The trial will be conducted in accordance with the Declaration of Helsinki (64th version, October 2013) and the guidelines for Good Clinical Practice (GCP). The in-and exclusion criteria are summarized in Table 1.

**Table 1:** In- and exclusion criteria.

Inclusion criteria	Exclusion criteria
Pathologically or cytologically confirmed ventral or pelvic malignancy, positive peritoneal metastasis of these cancer or positive exfoliated cells of these cancer were detected in peritoneal lavage fluid	Drug allergy history of TNF and its derivatives
Moderate or greater amount of peritoneal effusion. Definition of moderate ascites, ultrasonic examination of ascites in decubitus position (≥3 cm); accompanied with clinical symptoms (abdominal distension and discomfort)	Pregnant or lactating women, women of child-bearing age who were not willing to use contraception during the study period; or men are unwilling to use effective contraceptives during treatment and for the next year
Aged 18‐75 years male or non-pregnant or lactating women	Patients with severe or uncontrolled medical diseases and infections (including atrial fibrillation, angina pectoris, cardiac insufficiency, ejection fraction less than 50%, uncontrollable hypertension, body temperature > 38 °C, etc)
Bone marrow reserve function is good, blood routine meets the following conditions: white blood cell (>3.5 * 10^9/L), neutrophil (>1.5 * 10^9/L), platelet(>100 * 10^9/L), hemoglobin (>90 g/L)	Encapsulated peritoneal effusion
Good organ function, biochemical examination met the following conditions: ALT < 2.5 * ULN, AST < 2.5 * ULN, serum total bilirubin < 1.5 * ULN, serum creatinine < 1.5 * ULN	Severe hypoproteinemia
ECOG <= 1	HIV positive and active hepatitis
Voluntary signing of informed consent	Patients with severe or uncontrollable mental illness
	Any condition that is unstable or that could jeopardize the safety of the subject and their compliance in the study

### 2.1. Start trial

Inclusion, treatments started in Zhongshan Affiliated Hospital, Guangzhou University of Chinese Medicine by the end of 2021. Twenty patients will be enrolled in this trial.

### 2.2. Therapeutic method

After ascites was extracted as far as possible, the patients were treated by HIPEC combined with intracavity injection of rmhTNF-NC. The specific treatment methods were as follows: d1 and d4 were given cisplatin at a dose of 30 mg/m^2^, and the cisplatin and 2000 to 3000 mL 0.9% normal saline were added into a special treatment bag for body cavity thermoperfusion chemotherapy, and then the air in the bag was drained. The treatment bag was connected with the coelomic thermal perfusion therapy machine. After the liquid medicine in the treatment bag was heated to 43 °C, the machine was connected with the abdominal drainage catheter placed in the patient, and the coelomic circulation thermal perfusion therapy was carried out on the patient. D7 was mixed with 20 mL 0.9% normal saline, 10 mg dexamethasone and 3 million IU rmhTNF-NC, and injected into the abdominal cavity through abdominal drainage catheter. Between 2 and 4 hours after the infusion, the patient was asked to change his position every 15 minutes so that the solution was evenly distributed in his abdominal cavity.

### 2.3. Follow-up

During follow-up before and after the completion of HIPEC, physical examinations and measurements of white blood cell count, Hemoglobin, neutrophil count, blood platelet count, liver enzymes and serum creatinine levels were performed and then every 1 month for abdominal ultrasonography until the one-year point.

### 2.4. Primary and secondary objectives

Primary objectives: HIPEC effectiveness in the combined treatment of rmhTNF-NC in abdominal effusion caused by advanced OC will be analyzed. The response will evaluated according to the WHO criteria^[9]^ and will classify as complete response (CR): the abdominal effusion disappeared completely and lasted for more than 4 weeks, partial response (PR): more than 50% less effusion than before treatment (based on the maximum depth of effusion observed by B-ultrasound examination), stable disease (SD): fluid accumulation should be reduced by less than 50% or increased by less than 25% from before treatment (based on the maximum depth of fluid accumulation observed by B-ultrasound examination), and progressive disease (PD): Fluid accumulation increased by more than 25% compared with before treatment. Objective response rate (ORR) calculated by CR + PR. Disease control rate (DCR) calculated by CR + PR + SD. The evaluation of adverse reactions was divided into grade 0 to 4 according to NCI CT CAE 4.0.

Secondary objective: Quality of life (QoL), QoL was assessed with the European Organization for Research and Treatment for Cancer Quality of Life Questionnaire core 30 (EORCT QLQ-C30) by the dietician before HIPEC and 1 month after HIPEC.

### 2.5. Statistical analysis

SAS 9.4 statistical analysis software is used for calculation. Full analysis set (FAS) refers to the case that has used the research drug at least once and has at least 1 main index measurement data after treatment. For the case data of failure to observe the whole treatment process, the last observation data is used to carry forward to the final result last observation carried forward (LOCF) of the study. Cases never using the drug of this study will be excluded. Per Protocol Set (PPS) refers to the case that meet the inclusion criteria, do not meet the exclusion criteria, have valid baseline values, have good compliance, and do not violate the clinical trial protocol (e.g., do not use prohibited drugs, etc), on the basis of the FAS. Safety Set (SS) refers to the case that has used the study drug at least once after enrollment and have a record of safety indicators. Both the full analysis set and the protocol set were used in the efficacy analysis, and FAS results were the main results. Security analysis uses security data sets. The statistical or grade data is compared by *χ*^2^ test and two-category Logistic multiple regression (Enter method) to analyze the factors affecting the objective curative effects. *P* < 0.05 are considered as statistically significant difference.

### 2.6. Data monitoring

An independent data monitoring committee will appoint to ensure the quality of all investigator-initiated studies. The data Researcher will monitor all study data in accordance with GCP. Informed consent of selected individual participants will be examined. Validation of the source data (to verify that all data on the case report form are consistent with the source data) will be performed during field monitoring. The strength of this validation is related to the risk associated with the intervention being investigated, which is considered acceptable. For all the content involved, such as the informed consent, inclusion and exclusion criteria, and main results involved in the study.

## 3. Discussion

### 3.1. HIPEC

HIPEC is a treatment that fills the abdominal cavity with chemotherapy drugs through heating (43 °C), aiming to kill the remaining cancer cells in the abdominal cavity. HIPEC can effectively improve peritoneal drug penetration, increases the delivery of drugs to the peritoneal surface and eliminates residual microscopic peritoneal lesions more efficiently, potentially improving patient outcomes, compared to intravenous chemotherapy.^[10]^ HIPEC increases the penetration of chemotherapy into the peritoneal surface by heating and promotes the sensitivity of cancer to chemotherapy by blocking DNA repair. Heating also induces apoptosis and activates heat shock proteins that act as receptors for natural killer cells, inhibits angiogenesis and promotes protein denaturation to produce direct cytotoxic effects,^[11–15]^ while reducing systemic side effects associated with prolonged intraperitoneal assisted exposure. Moreover, there is emerging evidence that it can be used in both primary and recurrent cases in OC.^[16]^ In a meta-analysis, including two randomized controlled studies and 11 observational studies in 2019, the addition of HIPEC to cell destruction significantly improved overall survival (OS) in patients with OC. HIPEC may be one of the new therapeutic strategies for malignant disseminated peritoneal lesions.^[17]^ M06OVH-OVHIPEC suggested that HIPEC showed improvement in recurrence-free survival (RFS) (14.2 months vs 10.7 months) and median OS (45.7 months vs 33.9 months)in patients with FIGO stage III primary epithelial ovarian, fallopian tube, or peritoneal carcinoma, who receiving neoadjuvant chemotherapy (NACT) for extensive lesions to peritoneal cavity or unsatisfactory primary debulking surgery, and the percentage of patients with grade 3 or 4 adverse events was similar in the two groups.^[8]^ National Comprehensive Cancer Network guidelines indicate the addition of HIPEC with cisplatin (30 mg/m^2^) in patients with stage III OC who have received NACT during interval debulking surgery.^[18]^ HIPEC has been gradually applied in patients with colorectal cancer peritoneal metastases. One of the multicenter, randomized, open phase III trial of cytoreductive surgery plus HIPEC for colorectal peritoneal metastases (PRODIGE 7) showed no OS benefit,^[19]^ however, it may also be the result of inadequate selection of therapeutic parameters used during PRODIGE-7.^[20]^

### 3.2. Effective drug in the treatment of malignant abdominal effusion

Patients with OC is in advanced, once peritoneal metastasis occurred. Peritoneal metastasis due to external intestinal obstruction or mesenteric, intestinal muscle or nerve infiltration, are resulting in ascites, intestinal obstruction and other adverse symptoms, which eventually leading to death. With rapid development in modern biotechnology, even in Canada, the United States and other developed countries, various classes of medications can be effective, the 5-year survival rate of women diagnosed with OC is only 47%, and about 75% of patients will relapse. Therefore, it is difficult to completely cure and the overall prognosis is poor.^[21–23]^ There are currently effective medicines for malignant peritoneal effusions, including chemotherapy drugs, such as platinum drugs, paclitaxel, etc,^[24,25]^ modern traditional Chinese medicine preparations, such as elemene, etc,^[26]^ molecular targeted drugs, such as recombinant human endostatin, etc.^[7]^

### 3.2. Recombinant mutant human tumor necrosis factor for injection

Recombinant mutant human tumor necrosis factor for injection (rmhTNF-NC) is a modified rmhTNF, which the toxicity was reduced by 5 times and 10 times higher than that of the wild type.^[27]^ rmhTNF-NC belongs to a novel class of biologic, is mainly used in combination with chemotherapy for refractory advanced non-small cell lung cancer and non-Hodgkin’s lymphoma and also extensively used in the local treatment of advanced-stage thoracic and abdominal effusion. The major mechanisms of rmhTNF-NC involved in killing tumor cells mainly include, tumor cells undergo apoptosis or suppression via the mitochondrial pathway or the death receptor pathway. By activating inflammatory factors, it can have an effect on tumor vascular endothelial cells, cause endothelial cell damage or vascular dysfunction, cause thrombosis, block the blood flow of tumor tissue, and finally make it ischemic necrosis. Moreover, a certain concentration of rmhTNF-NC can inhibit the formation of neovascularization. It can regulate the immune function of the body and encourage other killer cells or T cells to inhibit and kill tumor cells. It can promote the expression of major histocompatibility complex (MHC) antigens of T cells, enhance the proliferation of T cells, and promote the production of interferon-γ (INF-γ), interleukin-2 (IL-2) and other factors, thereby motivating the secretion of immunoglobulin. It can promote the release of neutrophils from the bone marrow, enhance the phagocytosis function of neutrophils, enhance cytotoxic effect by increasing the production of peroxide anion, and promote the secretion of myeloperoxide, via the activation of bone marrow stromal cells to produce cerebrospinal fluid.^[28,29]^

Multiple studies have shown that rmhTNF-NC achieved excellent results in local treatment of advanced-stage thoracic and abdominal effusion. A prospective, open, single-arm, large-sample, multicenter phase IV trial involving 985 patients with rmhTNF-NC for monotherapy perfusion for malignant thoracic and abdominal effusion showed that the ORR of 916 patients after treatment was 62.44%, and the DCR was 97.27%. The ORR of malignant pleural effusion group was 70.52%, and that of malignant abdominal effusion group was 46.03%.^[30]^ A meta-analysis involving 694 patients from 12 studies revealed that pleural infusion of rmhTNF-NC combined with cisplatin increased ORR and improved QoL in patients with malignant pleural effusion.^[27]^

## 4. Conclusion

rmhTNF-NC and HIPEC with cisplatin is effective while treating patients with abdominal effusion caused by advanced OC, we will conduct a multicenter randomized controlled trial next, which may help expand the indication of HIPEC and is expected to provide an evidence-based basis for reducing mortality and improving QoL in patients with advanced OC.

### 4.1. Strengths and limitations of this study

This is a single center trial, which may limit the generalization of conclusions, consequently, multicentre clinical studies with a larger sample size will be required.

## Author contributions

This study was conceived and designed by HZ and LW, and coordinated by LW and JM. LW, JM,WZ, JW, CF and LL collected data, which were analyzed and interpreted by LW, WZ and JW. LW and WZ drafted the Article, and then critically revised it in conjunction with HZ, CF, and JM. All authors approved the final version.

**Conceptualization:** Jincheng Meng, Huatang Zhang, Wanshan Zhu.

**Investigation:** Lexia Wu, Jiaming Wu, Wanshan Zhu.

**Methodology:** Luzhen Li.

**Project administration:** Jincheng Meng, Huatang Zhang, Lexia Wu.

**Supervision:** Cantu Fang, Jincheng Meng, Huatang Zhang, Wanshan Zhu.

**Validation:** Luzhen Li.

**Writing – original draft:** Jiaming Wu, Lexia Wu.

**Writing – review &amp; editing:** Cantu Fang, Huatang Zhang.

## References

[1] Sung H Ferlay J Siegel RL. Global cancer statistics 2020: GLOBOCAN estimates of incidence and mortality worldwide for 36 cancers in 185 countries. CA Cancer J Clin. 2021;71:209–49. 33538338 10.3322/caac.21660

[2] Wang X Liu G Sheng N. Peptidome characterization of ovarian cancer serum and the identification of tumor suppressive peptide ZYX36-58. Ann Transl Med. 2020;8:925. 32953725 10.21037/atm-20-2018 PMC7475411

[3] Cripe J Tseng J Eskander R. Cytoreductive surgery and hyperthermic intraperitoneal chemotherapy for recurrent ovarian carcinoma: analysis of 30-day morbidity and mortality. Ann Surg Oncol. 2015;22:655–61. 25155402 10.1245/s10434-014-4026-6

[4] Wright AA Bohlke K Armstrong DK. Neoadjuvant chemotherapy for newly diagnosed, advanced ovarian cancer: society of gynecologic oncology and American society of clinical oncology clinical practice guideline. Gynecol Oncol. 2016;143:3–15. 27650684 10.1016/j.ygyno.2016.05.022 PMC5413203

[5] Armstrong DK Alvarez RD Bakkum-Gamez JN. NCCN guidelines insights: ovarian cancer, version 1. 2019. J Natl Compr Canc Netw. 2019;17:896–909. 31390583 10.6004/jnccn.2019.0039

[6] Li JX Shi YM An LY. Quality assessment of the guidelines for the management of malignant pleural effusions and ascites. World J Surg Oncol. 2020;18:331. 33308239 10.1186/s12957-020-02097-y PMC7733286

[7] Smolle E Taucher V Haybaeck J. Malignant ascites in ovarian cancer and the role of targeted therapeutics. Anticancer Res. 2014;34:1553–61. 24692682

[8] Van Driel WJ Koole SN Sikorska K. Hyperthermic intraperitoneal chemotherapy in ovarian cancer. N Engl J Med. 2018;378:230–40. 29342393 10.1056/NEJMoa1708618

[9] Zhang P Ai B. Solid tumor immunotherapy efficacy evaluation criteria. Int J Oncol. 2016;43:848–51.

[10] Eskander RN Tewari KS. Emerging treatment options for management of malignant ascites in patients with ovarian cancer. Int J Womens Health. 2012;4:395–404. 22927770 10.2147/IJWH.S29467 PMC3422105

[11] Tewari D Java JJ Salani R. Long-term survival advantage and prognostic factors associated with intraperitoneal chemotherapy treatment in advanced ovarian cancer: a gynecologic oncology group study. J Clin Oncol. 2015;33:1460–6. 25800756 10.1200/JCO.2014.55.9898 PMC4404424

[12] Ohno S Siddik ZH Kido Y. Thermal enhancement of drug uptake and DNA adducts as a possible mechanism for the effect of sequencing hyperthermia on cisplatin-induced cytotoxicity in L1210 cells. Cancer Chemother Pharmacol. 1994;34:302–6. 8033297 10.1007/BF00686037

[13] Panteix G Beaujard A Garbit F. Population pharmacokinetics of cisplatin in patients with advanced ovarian cancer during intraperitoneal hyperthermia chemotherapy. Anticancer Res. 2002;22:1329–36. 12168946

[14] Spratt JS Adcock RA Muskovin M. Clinical delivery system for intraperitoneal hyperthermic chemotherapy. Cancer Res. 1980;40:256–60. 6766084

[15] Van de Vaart PJ van der Vange N Zoetmulder FA. Intraperitoneal cisplatin with regional hyperthermia in advanced ovarian cancer: pharmacokinetics and cisplatin-DNA adduct formation in patients and ovarian cancer cell lines. Eur J Cancer. 1998;34:148–54. 9624250 10.1016/s0959-8049(97)00370-5

[16] Riggs MJ Pandalai PK Kim J. Hyperthermic intraperitoneal chemotherapy in ovarian cancer. Diagnostics (Basel). 2020;10(1):43. Published 2020 Jan 14. 31947647 10.3390/diagnostics10010043 PMC7168334

[17] Tsuyoshi H Inoue D Kurokawa T. Hyperthermic intraperitoneal chemotherapy (HIPEC) for gynecological cancer. J Obstet Gynaecol Res. 2020;46:1661–71. 32715605 10.1111/jog.14391

[18] Armstrong DK Alvarez RD Bakkum-Gamez JN. Ovarian cancer, version 2.2021, NCCN clinical practice guidelines in oncology. J Natl Compr Canc Netw. 2021;19:191–226. Published 2021 Agu 17. 33545690 10.6004/jnccn.2021.0007

[19] Quénet F Elias D Roca L. Cytoreductive surgery plus hyperthermic intraperitoneal chemotherapy versus cytoreductive surgery alone for colorectal peritoneal metastases (PRODIGE 7): a multicentre, randomised, open-label, phase 3 trial. Lancet Oncol. 2021;22:256–66. 33476595 10.1016/S1470-2045(20)30599-4

[20] Ceelen W. HIPEC with oxaliplatin for colorectal peritoneal metastasis: the end of the road? Eur J Surg Oncol (EJSO). 2019;45:400–2. 30392745 10.1016/j.ejso.2018.10.542

[21] Lheureux S Braunstein M Oza AM. Epithelial ovarian cancer: evolution of management in the era of precision medicine. CA Cancer J Clin. 2019;69:280–304. 31099893 10.3322/caac.21559

[22] Canadian Cancer Statistics Advisory Committee. Canadian cancer statistics 2017. Toronto, ON: Canadian Cancer Society. 2018.

[23] Van Baal JOAM van Noorden CJF Nieuwland R. Development of peritoneal carcinomatosis in epithelial ovarian cancer: a review. J Histochem Cytochem. 2018;66:67–83. 29164988 10.1369/0022155417742897 PMC5794203

[24] Nielsen M Graversen M Ellebæk SB. Next-generation sequencing and histological response assessment in peritoneal metastasis from pancreatic cancer treated with PIPAC. J Clin Pathol. 2021;74:19–24. 32385139 10.1136/jclinpath-2020-206607 PMC7788484

[25] Shigami H Fujiwara Y Fukushima R. Phase III trial comparing intraperitoneal and intravenous Paclitaxel Plus S-1 versus cisplatin plus S-1 in patients with gastric cancer with peritoneal metastasis: PHOENIX-GC trial. J Clin Oncol. 2018;36:1922–9. 29746229 10.1200/JCO.2018.77.8613

[26] Zhu J Li B Ji Y. β-elemene inhibits the generation of peritoneum effusion in pancreatic cancer via suppression of the HIF1A-VEGFA pathway based on network pharmacology. Oncol Rep. 2019;42:2561–71. 31638231 10.3892/or.2019.7360 PMC6826333

[27] Fu T Lin Y Zeng Q. Thoracic perfusion of recombinant mutant human tumor necrosis factor (rmhTNF) can be considered as a good adjunct in the treatment of malignant pleural effusion caused by lung cancer. BMC Pulm Med. 2020;20:175. 32552897 10.1186/s12890-020-01210-x PMC7301477

[28] Wang H Gong T Zhou S. Association of TNFAlP3 gene polymorphisms with prognosis of patients with esophageal squamous cell carcinoma. Ann Oncol. 2019;25:325–9.

[29] Guo Q Shi X. Recombinant mutant human tumor necrosis factor versus pleural perfusion of cisplatin in the treatment of malignant pleural effusions:a systematic review. Chin Pharm. 2018;29:839–42.

[30] Qin SK Liu XF Ma J. Prospective, multi-center clinical study of rmhTNF-NC injection in the treatment of Chinese malignant pleural effusion or perioneal effusion patients. Chin Clin Oncol. 2016;21:577–84.

